# Cost–Utility Analysis of 3-Month Telemedical Intervention for Heart Failure Patients: A Preliminary Study from Poland

**DOI:** 10.3390/healthcare12131360

**Published:** 2024-07-08

**Authors:** Piotr Wańczura, David Aebisher, Mateusz Wiśniowski, Marek Kos, Hubert Bukowski, Dominik Golicki, Andrzej Przybylski

**Affiliations:** 1 Department of Cardiology, Medical College of Sciences, The Rzeszów University, 35-310 Rzeszow, Poland; a_przybylski-65@wp.pl; 2 The Ministry of Internal Affairs and Administration Hospital, 35-111 Rzeszow, Poland; m.m.wisniowski@gmail.com; 3 Department of Photomedicine and Physical Chemistry, Medical College, University of Rzeszów, 35-310 Rzeszow, Poland; daebisher@ur.edu.pl; 4 Department of Public Health, Medical University of Lublin, 20-400 Lublin, Poland; marekkos@op.pl; 5 Institute of Innovation and Responsible Development, 02-621 Warsaw, Poland; h.bukowski@innowo.org; 6 Department of Experimental and Clinical Pharmacology, Medical University of Warsaw, 02-097 Warsaw, Poland; dominik.golicki@wum.edu.pl

**Keywords:** cost–utility of telemedical intervention, quality of life, heart failure, cost–utility analysis, short-lasting telemedical model

## Abstract

Heart failure (HF) is a common clinical syndrome in which the cardiac systolic and/or diastolic functions are significantly insufficient, resulting in an inadequate pump function. Currently, it is one of the leading causes of human death and/or hospitalization, and it has become a serious global public health problem. Approximately 1.2 million people in Poland suffer from HF, and approximately 140,000 of them die every year. In this article, we present the result of telemedicine intervention and its cost-effectiveness in a group of patients from a pilot program on telemedicine and e-health solutions reducing social inequalities in the field of cardiology. Based on the EQ-5D-5L questionnaire administered in the beginning of the project and after approximately 3 months, used for the health state utility values calculation, cost estimates of the project, and inclusion of supplementary data, the economic rationale behind telemedical intervention in HF patients using a cost–utility analysis was corroborated. The choice of a 3-month project duration was due to the top-down project assumptions approved by the bioethics committee. The average improvement in health state utility values was statistically significant, implying a 0.01 QALY improvement per patient. The cost of the telemedical intervention per QALY was well within the official limit adopted as a cost-effective therapy measure in Poland.

## 1. Introduction

Heart failure (HF) is a common clinical syndrome in which the cardiac systolic and/or diastolic functions are significantly insufficient, resulting in an inadequate pump function. Currently, it is one of the leading causes of human death and/or hospitalization, and it has become a serious global public health problem [1]. Approximately 0.9% of the global population suffer from HF; this proportion increases significantly with the aging population [2].

The data concerning the HF epidemiology in Poland are even more disturbing. Approximately 1.2 million people in Poland suffer from HF, and approximately 140,000 of them die every year [3]. In 2018, HF patients were 3.2% of the total population, 11.7% of the 60+ population, and almost 29.8% in the age group over 80 years old [4]. In addition, heart failure is the most common direct cause of death in Poland, with over 40.5 thousand deaths in 2018, accounting for 9.8% of all deaths.

The burden of this disease on the public healthcare system is also considerable. This fact results directly from the common prevalence of the disease, but also on the fact that Poland is the leader among the Organization for Economic Co-operation and Development (OECD) countries when it comes to the number of hospitalizations related to the treatment of heart failure. In 2021, there were 459 hospitalizations per 100,000 citizens, compared with an average of 206 hospitalizations for 28 OECD countries [5].

Multidisciplinary co-operation between primary care physicians, nurses, and cardiologists is the recommended form of heart failure patient care. In the field of cardiology, telemedicine solutions have become an extension of this care in the population dimension [6,7]. Effective telemonitoring programs need real-time access to relevant patient data, adequate human resources to run the project and manage this data, and the right target group for which the telemedicine intervention will not only be effective but also provide the necessary level of knowledge to consolidate health-promoting behaviors in the future [8].

The current state of knowledge allows for this statement, that combining the standard treatment of heart failure with telemedicine solutions allows us to achieve higher survival rates and has a positive impact on quality of life (QoL). On the other hand, the above-mentioned solutions inevitably require a higher expenditure on the part of the payer and effort on the part of the patients [9]. The above assumptions became the basis for searching for new technological solutions that will not only enable remote disease management, but also meet the criterion of cost-effectiveness compared to direct methods. In over sixty studies and numerous meta-analyses from 2000 to 2019, the effectiveness of telemonitoring in patients with HF was an assessed clinical effect. Most of them (63%) clearly confirmed the importance of the above-mentioned solutions in the context of reducing mortality and the unplanned need to use healthcare facilities. All seven meta-analyses mentioned above clearly demonstrated a reduction in mortality and the risk of hospitalization due to exacerbations of heart failure.

Details on the research review are provided in Annex 3 [8]. Taking into account the ongoing development of telemedicine solutions, no one has any doubts, that a well-designed and implemented project in this area has a positive impact on the quality of healthcare. The implementation of such a model itself is not only time-consuming, but also requires the precise definition of specific goals through the prior estimation of the needs and profitability map, so that the program can be implemented in the pilot phase.

Even the period of effective monitoring itself is questionable and its optimal duration has not been conclusively established [9,10]. A longer active phase of the model is not necessarily better than a short but intensive pattern. There are sources that confirm effectiveness of a 3-month real-time, high-intensity exercise program for congestive heart failure (CHF) patients who are either unable or unwilling to participate in standard outpatient cardiac rehabilitation [11].

Telemedicine offers a chance to improve the current dire state. In the described pilot project “Reducing social inequalities in health through the use of telemedicine and e-health solutions” in the field of Cardiology for approximately 12 weeks of observation, a total group of 429 patients from five voivodeships, mainly from the south-eastern part of Poland, was recruited. Each project participant was encouraged to use a specific model of telemedicine care, including daily monitoring in real time (via the GSM—Global System for Mobile Communications—network). It is worth emphasizing once again that over 100,000 telemedicine tests were carried out in the active phase of the study.

The use of the telemedicine model described above resulted in the effective optimization of the pharmacological profile of most patients. Favorable effects were achieved as a result of the improved co-operation between primary care physicians and cardiology specialists who remotely co-ordinated each of the included centers. In this way, during the study period, it was possible to minimize the disproportion in access to specialized cardiological care, and 575 changes in the treatment regimen of patients were implemented during these 12 weeks.

This took place initially during the first teleconsultation with a cardiologist, and then during a teleconsultation with a primary care physician and during a second teleconsultation with a cardiologist.

With the use of the EQ-5D-5L questionnaire administered in the beginning of the project and after approximately 3 months, cost estimates of the project, and inclusion of supplementary data, this publication aims to corroborate the economic rationale behind telemedical intervention in HF patients using a cost–utility analysis (CUA). EQ-5D-5L stands for EuroQol—5 dimensions–5 levels and is a generic instrument developed in Europe and widely used the quality-of-life evaluation in different diseases. The EQ-5D-5L descriptive system is a preference-based health-related quality-of-life (HRQL) measure with one question for the five dimensions included (mobility, self-care, daily activities, pain–discomfort, anxiety, and depression) and each dimension has five levels (no problems, slight problems, moderate problems, severe problems, and extreme problems). The mentioned questionnaire is considered very intuitive and easy to complete. 

The rationale for choosing a CUA over other forms of economic evaluation is twofold. Firstly, it takes into account the quality of life, not only its length. Secondly, the use of a standardized, generic measure, such as the quality-adjusted life year (QALY) used in this analysis, enables economic comparisons across different disease areas with very different clinical outcomes. The CUA utilizes the real-life cost difference of standard therapy against the assessed innovative intervention to calculate the cost of an additional QALY, i.e., the incremental cost–utility ratio (ICUR) established by the difference in total cost expressed in monetary terms per QALY. This enables decision-makers to economically assess the overall economic effectiveness of an intervention to support them in their budget allocation across the whole spectrum of disease areas, as well as different types of interventions. QALYs measure the length of life adjusted for its quality, resulting in what can be thought of as an equivalent of years of life in full health. For various health conditions, the conversion factor of a year spent in a given state to an equivalent of a year in full health is referred to as the health utility [12]. The comparability is a crucial aspect that makes the CUA routinely used in public payers’ decisions across the world [13]. Therefore, this economic evaluation method was deemed appropriate also in the case of the economic analysis of telemedical intervention in HF patients.

## 2. Materials and Methods

In this research, we used data accumulated from the project entitled “The impact of the telemedicine model on the change in clinical characteristics, the risk of recurrence of hospitalization and the maintenance of optimal pharmacotherapy in the group of patients with chronic heart failure.” This project was a multicenter, open, non-controlled trial carried out by the University of Rzeszów, Poland. The consent of the ethics committee for trial number 2023/06/0032 was obtained on 21 June 2023. The data points were collected between June 2023 and December 2023. The project included 429 patients with history of chronic heart failure remaining under control of primary care physicians, whose stage of heart failure was an overwhelming majority of class II or III according to the New York Health Association (NYHA) classification. The inclusion criterion was previously diagnosed heart failure, that was confirmed by a cardiologist on the day of recruitment. All the patients were initially assessed on the NYHA scale and the left ventricle ejection fraction was not obligatory to include the patient in the study unless the diagnosis of the disease was questionable by the residing cardiologist. The recruitment took place only among outpatients from family doctors’ offices who were preselected based on their previous medical history, including symptoms and treatment of chronic heart failure. Finally, all participants were recruited from 14 primary care units from 5 voivodeships, mostly considered social exclusion areas. Podkarpackie Voivodeship inhabitants comprised 52.7% of the patients recruited. The recruited patients had to declare their willingness to participate in the project for a period of not less than 3 months. The lack of this declaration was the only exclusion criterion for recruitment.

**Figure 1:**
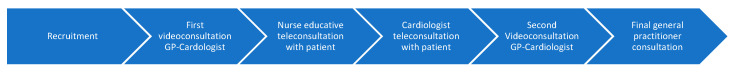
Basic scheme of the order of patient teleconsultations conducted in this project.

For a period of ca. 3 months, patients were monitored daily in real time (via the GSM—Global System for Mobile Communications—network) in terms of body weight and blood pressure using an electronic scale and an electronic blood pressure monitor (measurements 2 times a day plus times on request). After initial recruitment for the scheduled 3-month period of the study agreed upon in advance, patients were encouraged to undergo regular measurements of body weight and blood pressure using the same type and model of electronic blood pressure monitor (measurements 2 times a day plus times on request) and electronic scale (body weight once a day in the morning directly after using the bathroom). The patients did not incur any costs and all electronic devices were loaned to the patients for study period for free. The telemonitoring was conducted by a selected center open 24 h a day, seven days a week. In the event of any significant abnormalities as defined individually for each patient (e.g., abnormal weight gain, or high or significantly low blood pressure values), a telephone intervention was performed. The paramedic, or, in special cases, physicians, were able to modify pharmacological treatment for that particular day or propose a permanent change. In the event of abnormal weight gain, or high or low blood pressure values defined as atypical compared to the baseline, a telephone intervention, often combined with pharmacological modification, was performed by a paramedic or specialist physician through a selected monitoring center. Teleconsultations with medical personnel, including two on-site personal consultations with family doctor, two video consultations between GP and cardiologist, and one teleconsultation between the patient and responsible cardiologist, took place for each patient according to the scheme presented in Figure 1.

During the active phase of the project, over 100,000 tests were carried out. Different kinds of detected abnormalities have resulted in 575 changes in permanent medical treatment.

### 2.1. Resulting Study Pool and Variables

The research material was collected from a group of 349 patients from the total sample of 429 patients. These were the patients that fully completed the EQ-5D-5L questionnaire at the beginning of the program (recruitment) and at the end during the final general practitioner consultation. One hundred and nineteen patients from the total sample size of 429 did not fill out the initial or final EQ-5D-5L questionnaires or left it incomplete. Only patients with complete data on costs and effects (utility), at the inclusion to the project and at three months, were included in the CUA

The primary endpoint was the incremental cost–utility ratio (ICUR) established by the difference in mean total cost expressed in Polish zloty (PLN) per quality-adjusted life year (QALY) between basic supportive care (BSC) and basic supportive care aided with telemedicine (BSC + T). Basic supportive care was defined as healthcare services received during the three-month period preceding inclusion in the project, with health state utility values (HSUVs) calculated using the EQ-5D-5L questionnaire filled out by each patient at the point of inclusion. Basic supportive care aided with telemedicine was defined as the period of ca. 3 months following inclusion in the project with HSUVs calculated using the EQ-5D-5L questionnaire filled out during final general practitioner consultation.

The statistical significance of QoL differences was established first to prove that CUA is a suitable comparison technique between BSC and BSC + T.

To estimate QALYs, a Polish EQ-5D-5L-based directly measured value set [11] was used for this calculation. Total BSC cost computations used data from the initial questionnaire on all-cause hospitalization, ambulatory specialist and general practitioner visits in the three-month period prior to recruitment, and data on the medication use at the point of recruitment.

Total BSC + T costs used three sources of information. The first source was data from the final questionnaire on medications completed at the final general practitioner consultation. The second source was data from hospitalization during the 3 months of intervention gathered during the project’s operations. The third source was the cost of telemedical intervention for each patient calculated based on the official accounting data for the project.

To compute the cost of healthcare services for hospitalization, and ambulatory specialist and general practitioner visits, population data available in the public payer’s databases were obtained from the Podkarpackie Branch of the National Health Fund as over half of the studied pool (61.0%) was treated in the region. The average cost of those services in the first three quarters of 2023 was assumed as the mean cost per service for both BSC and BSC + T. Medicine costs were obtained from an official list of medicines reimbursed in Poland as of 1 September 2023 [14]. Data on the most popular medicine and its dosage in each of the chosen heart failure pharmaceutical groups were used for this calculation.

Basic supportive care costs in the three months preceding inclusion in the program were calculated basing on the information gathered during recruitment to the program, i.e., the number of healthcare services provided during that period, as well as information on administered pharmacotherapy. The average costs of hospitalization, outpatient specialist care, and primary care, and the proportion of other forms of healthcare services were obtained from public payer’s data from the Podkarpackie Voivodeship in the first to third quarter of 2023. The cost of pharmaceuticals was calculated based on the median dose and official cost of most popular drug in each pharmaceutical category.

### 2.2. Data Analysis

Baseline characteristics of the study pool utilized descriptive statistics. For continuous data with normal distributions, means and standard deviation (SD) were used. Categorical data employed absolute numbers and percentages.

The analysis uses repeated-measures design. It involved measuring the same variables taken on the same subjects at two different points in time. Therefore, a paired t-test was deemed sufficient to investigate whether true mean difference between the paired samples is zero. A *p*-value less than or equal to 0.05 was considered indicative for statistical significance. Statistical analysis was performed using StatsDirect software, version 2.8.0.

### 2.3. Ethics

The study was conducted in accordance with the Declaration of Helsinki and the laws and regulations applicable in Poland. Written approval from the appropriate Ethics Committees was obtained.

Patients were informed about the purpose of the project and their right to refuse or discontinue involvement. Each patient had to provide written informed consent on voluntary basis. Anonymity of patients was preserved throughout the analysis process.

## 3. Results

### 3.1. Descriptive Characteristics

Descriptive characteristics at the point of inclusion for all 346 patients in the study pool are presented in Table 1. The mean age equaled ca. 70 years. Almost 60% of the study group consisted of male patients, compared to the male predominance in the Podkarpackie Voivodeship HF population of 53.3%. More than half of the study pool was categorized as class II NYHA. Among the most prevalent comorbidities—over 30% of the study pool—were hypertension followed by dyslipidemia and diabetes. Ca. 30% of all patients had a myocardial infarction history. Almost 25% underwent angioplasty procedures in the past. Beta-blockers, angiotensin-converting enzyme inhibitors, loop diuretics, oral anticoagulants, and thiazide diuretics were the most prevalent type of pharmaceuticals used in the study pool.

#### 3.1.1. QALY Difference between Basic Supportive Care and Basic Supportive Care Aided by Telemedicine

The mean health state utility values (HSUVs) (Table 2) calculated for each patient in the initial EQ-5D-5L questionnaire at the point of recruitment equaled 0.853 (0.176). At the point of the final general practitioner consultations, the mean HSUVs calculated using the final EQ-5D-5L questionnaire equaled 0.923 (0.123). The average improvement in HSUVs reached 0.070 (95% CI 0.090 to 0.050) and was statistically significant with *p* < 0.0001 using the paired t-test. It is worth noting that the average length of time between the completion of the initial and final questionnaire was 128 days, which is in line with the 3 months adopted in project planning.

Over the course of the study, 59.4% of patients reported improvements, while 28.0% reported a deterioration in their health. No change in health state was reported by 12.6% of patients.

The relation between HSUV changes and a number of indicators using a multivariate analysis has been somewhat confounding. We have not found any changes in HSUV dependance on telemedical intervention intensiveness indicated by the number of pharmacotherapy modifications and number of telemedical examinations. The female sex has been associated with a statistically significant improvement in HSUVs. Therefore, it may be stated that telemedical intervention for HF patients is better suited for women. Among comorbidities, previous cardiac procedures, and selected health indicators, only the initial NYHA class has been found to be statistically significant. However, in a surprising fashion, the higher the initial NYHA class, the smaller improvement in HSUVs (Table 3).

#### 3.1.2. Cost Comparison between Basic Supportive Care and Basic Supportive Care Aided by Telemedicine

The project cost per three months was calculated using official accounting information. The only modification compared to the real-world data was the technical assumption that the service life of scales and blood pressure monitors equals one year, instead of the 3 months they have been used for during the project. According to this estimate, the cost of 3 months of telemedicine care would amount to about PLN 1162 (Table 4).

The telemedical intervention resulted in substantially less hospitalization. There were 3 all-cause hospitalizations during the 3 months of the project (with only one hospitalization due to HF) compared to 30 hospitalizations for 3 months preceding inclusion in the project. As a result (Table 4), the cost of hospitalization in BSC + T decreased by a calculated 221.2 thousand PLN (88.4%). Pharmaceutical costs increased by 22.2 thousand PLN (16.9%), which is the result of the pharmacotherapy modifications implemented during the project. Outpatient specialist care and primary care was replaced with extensive telemedical care. Therefore, their costs fell to 0 PLN, while the cost of telemedical intervention equaled 538.8 thousand PLN. This is a substantial number that results in the total BSC + T costs being notably higher than BSC by 329.4 thousand PLN (84.0%) (Table 5).

### 3.2. Cost–Utility of Telemedical Intervention

The change in HSUV implies that the number of quality-adjusted life years improved after 3 months of the project with an average addition of 0.01 QALY per patient. The total QALYs over the 3-month period were estimated using the area-under-the-curve method. The trapezoidal method was chosen as the more conservative method [15].

The mean cost difference between the BSC and BSC + T equaled 401.21 PLN per patient. This implies that the cost per QALY equaled 45,648.55 PLN. This number is well within the limit of the threefold GDP adopted by the Polish Agency for Health Technology Assessment and Tariff System as a cost-effective therapy measure (190.4 thousand PLN for 2023).

**Figure 2:**
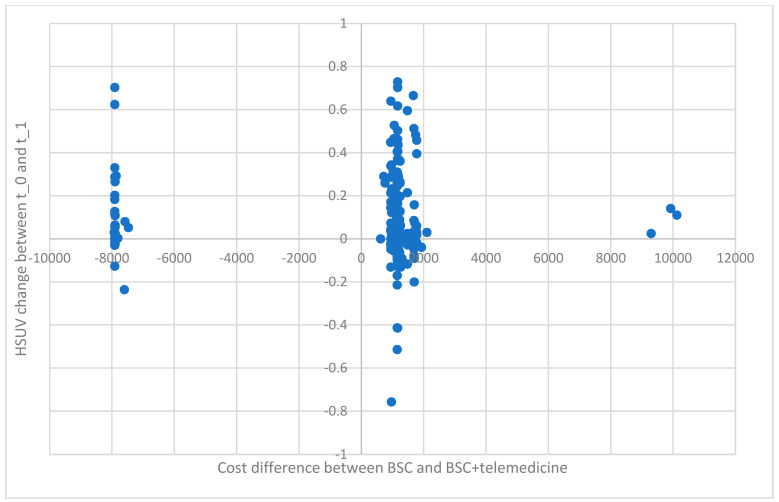
Individual HSUV change in relation to the difference in the cost of BSC and BSC + T.

Analyzing the individual HSUV changes against the difference in BSC and BSC + T costs (Figure 2), one can clearly see the role hospitalization plays in the total cost of healthcare. Among patients who underwent hospitalization 3 months before inclusion in the program, there were no rehospitalizations. A predominant part of that group (90.0%) increased their health utility.

## 4. Limitations

The main limitation of this analysis is its non-standard design. We compared the same pool of HF patients for an equal period before and after inclusion in the project. Therefore, there was no control group. This is a non-standard approach used relatively sparsely in telemedicine intervention in HF patient studies [16]. This approach is characterized by several shortcomings.

The main flaw of such a design is the time-inconsistency between the comparator and benchmark groups. The lack of use of a control group in the analysis can result in the index utilities for health-related quality of life as the HF severity increases with age [17]. However, it is not clear what the exact direction of the age-related change in health-related quality of life (HRQL) is for HF patients. Some studies claim stable HRQL scores with aging [18], while others report an improvement after one year [19]. The time horizon for the analysis may have been too short to observe an aging-related change in quality of life. However, this fact needs further investigation.

Furthermore, the time-inconsistency can result in seasonal effects of quality-of-life measures between the compared groups. A parallel analysis of the comparator and benchmark groups in the same period would result in findings independent from seasonality. However, it needs to be added that, typically, these measures rise during spring and summer and decrease in autumn and spring [20]. However, the BSC + T group was analyzed between June and December, while the BSC group was analyzed between March and September, which should work in a different direction than the observed outcomes.

We did not investigate various factors, such as the NYHA class, tobacco exposure, low ventricular ejection, history of hospitalization, comorbidity, polypharmacy, and duration of heart failure, that may be related to quality of life in heart failure patients, as the analyzed group consisted of the same individuals, examined at different points in time. A three-month difference between examinations was assumed to cause no notable changes in most of those factors, while others, such as the improvement in NYHA class, is an expected outcome of the telemedical intervention, that directly feeds into the enhancement of the HRQL. The assumption of such effects being unimportant is a notable constraint of the study.

Capturing the total cost of basic supportive care and basic supportive care aided with telemedicine is another limitation. The study assumed there were no additional HF outpatient specialist care and general practitioner visits beyond those prescribed in the telemedical intervention. This is a common-sense assumption based on the intensive healthcare services provided to the study pool patients. However, one cannot preclude outpatient specialist care and general practitioner visits during the 3-month periods. On the other hand, the BSC counts only one hospitalization outpatient specialist care and general practitioner visits used for calculation. A higher number of those services for some patients may be possible. Other than that, the short time span of the analysis should not directly influence the computation of the hospitalization, medication, and outpatient care costs. Real-world data on the usage of those medical services have been gathered. While the cost of single services is independent from the length of the analysis, hospitalization costs have been analyzed based on the mean cost from the public payers’ database. The same applies to outpatient care costs. Medications costs have been calculated based on the individual pharmacotherapy at the point of inclusion and at the time of the final visit, and the real-world prices of the medications.

## 5. Discussion

The role of telemedicine in HF patients has been widely studied. Synthesized findings from literature reviews indicate that telemedicine interventions may improve outcomes in heart failure patients [21,22,23]. Research has shown a potential reduction in heart-failure-related hospitalizations, morbidity, and mortality using telemonitoring programs in a significant number of publications [24,25,26]. However, the economic rationale for telemedicine in HF treatment has been analyzed to a much smaller extent and the findings from current research studies are often inconclusive [27,28]. One such analysis found a significant reduction in hospitalization costs but only in the third year after the telemedical intervention. At the same time, ambulatory expenses grew while medical aids and appliance costs were small and insignificant [29]. Another study concerned a 20-month trial carried out on Australian patients [30]. A reduction in costs from the readmission avoidance per patient for the 6 months of participation as well as for the projected 12-month real-world model was observed. Although remote monitoring is a very promising addition to standard cardiological care, there are several issues that remain a challenge. Some of these challenges are the standardization of the devices, the data, and monitoring time, and, finally, correctly identifying the target group among HF patients that would benefit the most [31].

As much as the cost–utility or cost-effectiveness analysis for telemedical intervention in HF patients is concerned, it is sparse or unconvincing [32]. A cost–utility study for heart failure patients with implantable defibrillators [33] established that remotely monitored patients gained 0.065 QALYs more than the control arm over 16 months, with a significant amount of cost savings. Therefore, remote monitoring was considered a cost-effective solution. The same conclusion concerned a 1-year study for Danish HF patients. The 1-year adjusted QALY difference was statistically insignificant. However, the adjusted difference in costs showed a reduction in total healthcare costs by 35%, supporting the economic rationale behind telemedical intervention in HF patients.

There are a few cost–utility studies for HF patients concerning the efficacy of telerehabilitation. One must bear in mind that these interventions differ substantially from the teleintervention studied in this article. According to one analysis [34] for Polish HF patients, telerehabilitation appears to be less costly and as effective for the healthcare provider as traditional center-based rehabilitation. A similar result was seen in Australia [35].

Notable examples of cost–utility studies concern analyses based on modelling. One study [36] used a cost–utility analysis model simulating hospitalizations and mortality in patients with HF to estimate outcomes and costs. The likelihood of the telemedical program being considered cost-effective was 90%. Another simulation study [37] showed that the probabilities of HF telemedical intervention cost-effectiveness were greater than 85%, both for the baseline and for alternative scenarios. In view of the relatively sparse and inconclusive examples in the literature, this article aims to provide an additional real-world analysis.

The statistically significant HSUV increases after participating in the project should not be attributed to the fact that there were notably less hospitalizations. Although the impact of hospitalization in the health-related quality of life of heart failure patients has not been analyzed extensively, one study disproves the notion that health-related quality of life decreases after hospitalizations [38]. Using patient-level data, the calculated mean difference between the index utilities for health-related quality of life measurement pre and post hospitalization was found to be 0.020 [95% CI: −0.020, 0.059]. The authors concluded that there is no significant evidence of a difference between utility pre and post hospitalization.

The number of hospitalizations was notably lower than in the 3 months before patient recruitment (3 and 30 hospitalizations, respectively). In view of readmission being a common event for HF patients, with the first few weeks after discharge from hospital being the highest risk period, between 20 and 30% of patients are readmitted within 30 days, rising to 50% at 6 months [39]; the reduction in hospitalization is even more substantial.

The assumptions of the BSC + T costs were made based on the accounting information for the project. The cost of the scaled-up telemedical intervention would most probably be lower than in the pilot project. This would result from the lower costs per person for the recruitment and communication activities as well as the optimization of the medical device life cycle. The medical personnel cost might also decrease as a scaled-up program should incorporate improved organization, telemedical intervention knowhow improvement, and overall time-efficiency gains, especially in terms of using information technology systems.

An important factor in this study is the sustainability of improved health utility states. This study seeks to provide further clinical follow-up consisting of changes in quality of life by additional EQ-5D-5L questionnaires 12 months after recruitment. This will not affect cost-effectiveness, but it may answer the question of whether the tested telemedicine model was carried out long enough to consolidate the effect of pro-health behaviors. The estimation of the real costs of the model allows us to plan the population use of the presented solutions in the context of the population control of a larger group of potential beneficiaries. Modern E-solution protocols have become the standard approach in many developed societies. Although the optimal length of telemedicine projects has not been clearly defined, their extension does not necessarily result in an improvement in clinical effects [40]. It is, therefore, highly probable that a longer time horizon would not see the HSUVs persist [41]. Considering the aforementioned limitations, it is worth stating that the relatively short analysis timespan is coherent with the analysis of the same study pool before and after the telemedical intervention. It seems that most of the health state changes detected in the 3-month period should stem from the modification of the therapy, while non-medical factors should remain relatively unaltered. In other words, observing meaningful health state changes even in such a short time should be considered a direct result of the intervention, and, to a lesser extent, an effect of other factors, such as aging, comorbidities, lifestyle modification, etc.

Health state changes after a medical intervention in HF patients have already used such short timespans with good results. Xie et al. [42] use, for example, a shorter version of the EQ-5D questionnaire (EQ-5D-3L) to assess the efficacy of dapagliflozin. The analyses of medical intervention carried out for a similar period using the EQ-5D-5L questionnaire to assess patients’ health states have been implemented in other morbidities as well [43,44,45].

Future research should concentrate on the validation of the findings, including longer-term studies to assess the sustainability of the observed improvements and larger-scale trials to confirm the efficacy and cost-effectiveness of telemedical intervention. It seems that further studies with a wider population range, as well as different wavelengths of the active observation phase, would be needed to determine the optimal target to be used more and more often in modern cardiology telemedicine solutions.

## 6. Conclusions

The use of the lasting 3-month telemedicine model, i.e., daily measurements of body weight and blood pressure values assessed by an algorithm in real time, together with an additional cardiology and general practitioner consultation, resulted in a statistically significant (*p* < 0.0001) increase in health state utility values of 0.070 (95% CI 0.090 to 0.050).

This HSUV implies that the number of quality-adjusted life years has improved in only 3 months of the project‘s duration with an average addition of 0.01 QALY per patient. The cost difference between the basic supportive care and basic supportive care aided with telemedicine equaled 401.21 PLN per person. This implies that the cost per QALY equaled 45,648.55 PLN. This number is well within the limit adopted by the Polish Agency for Health Technology Assessment and Tariff System as a cost-effective therapy measure.

## Figures and Tables

**Table 1:** Population baseline characteristics.

	n = 346
Age, mean (SD)	69.8 (10.3)
Female gender, n (%)	138 (39.9%)
Body mass in kg, mean (SD)	85.2 (16.0)
DBP in mmHG, mean (SD)	70.9 (10.9)
SBP in mmHG, mean (SD)	133.5 (22.2)
HR, mean (SD)	78.5 (12.2)
NYHA class, n (%)	
I	27 (7.8%)
II	214 (61.8%)
IIII	87 (25.2%)
IV	18 (5.2%)
Comorbidities, n (%)	
Hypertension	261 (75.4%)
Dyslipidemia	196 (56.6%)
Diabetes	105 (30.3%)
Myocardial infarction history	103 (29.8%)
Atrial fibrillation	100 (28.9%)
Nicotinism	36 (10.4%)
Hypothyroidism	35 (10.1%)
Chronic kidney disease	34 (9.8%)
Valvular defects	33 (9.5%)
Asthma	30 (8.7%)
Cancer	27 (7.8%)
Stroke history	23 (6.6%)
Thrombosis	22 (6.4%)
Obstructive pulmonary disease	21 (6.1%)
Other thyroid diseases	19 (5.5%)
Hyperthyroidism	9 (2.6%)
Cardiomyopathy	6 (1.7%)
Previous cardiac procedures, n (%)	
Angioplasty	85 (24.6%)
Coronary artery bypass grafting	24 (6.9%)
Implanted cardioverter	18 (5.2%)
Medications, n (%)	
Beta-blocker	292 (84.4%)
Angiotensin-converting enzyme inhibitor	215 (62.1%)
Loop diuretic	161 (46.5%)
Oral anticoagulant	133 (38.4%)
Thiazide diuretic	121 (35.0%)
SGLT2 inhibitor	86 (24.9%)
P2Y12 inhibitor	34 (9.8%)
Sacubitril/valsartan	13 (3.8%)

**Table 2:** Health state utility values at inclusion in the project and at its completion (n = 346).

Variables	Initial HSUV	Final HSUV	HSUV Change
Mean	0.853	0.923	0.070 *
Standard deviation	0.176	0.123	0.180
Upper quartile	0.97	1	0.11
Median	0.925	0.952	0.023
Lower quartile	0.795	0.873	−0.018
Centile 95	1	1	0.448
Centile 5	0.497	0.752	−0.116

* statistically significant at *p* < 0.0001.

**Table 3:** Multivariate regression analyses of HSUV changes.

**Variables**	**Coefficient**	**SD**	***p*-Value**
Telemedical intervention intensiveness			
Number of pharmacotherapy modifications	0.0126	0.0068	0.0639
Number of telemedical examinations	0.0001	0.0001	0.2985
Constant	0.0366	0.0226	0.1051
Patients’ basic characteristics			
Age in years	0.0004	0.0010	0.6469
Sex (female = 1)	0.0439	0.0207	0.0349 *
Constant	0.0219	0.0672	0.7455
Comorbidities, previous cardiac procedures, and selected health indicators			
Hypertension	0.0251	0.0220	0.2548
Dyslipidemia	−0.0097	0.0165	0.5596
Diabetes	−0.0203	0.0166	0.2217
Myocardial infarction history	0.0214	0.0205	0.2976
Atrial fibrillation	−0.0056	0.0174	0.7479
Nicotinism	0.0124	0.0244	0.6118
Hypothyroidism	−0.0192	0.0244	0.4319
Chronic kidney disease	−0.0080	0.0259	0.7567
Valvular defects	−0.0499	0.0264	0.0597
Asthma	0.0069	0.0276	0.8024
Cancer	0.0149	0.0277	0.5906
Stroke history	−0.00108	0.0288	0.9702
Thrombosis	−0.0277	0.0299	0.3543
Obstructive pulmonary disease	0.0167	0.0352	0.636
Other thyroid diseases	0.0112	0.0329	0.7346
Hyperthyroidism	0.0023	0.0502	0.9631
Cardiomyopathy	0.0564	0.0621	0.3652
Angioplasty	0.0062	0.0205	0.763
Coronary artery bypass grafting	−0.0195	0.0296	0.5096
Implanted cardioverter	0.0047	0.0359	0.8955
NYHA class at inclusion	−0.0487	0.0122	0.0001 *
Hospitalization in previous 3 months	0.0391	0.0262	0.1371
Constant	1.0111	0.0359	<0.0001 *

* statistically significant at *p* < 0.05.

**Table 4:** Breakdown of telemedical intervention costs per person in PLN.

Type of Action	Cost in PLN
Recruitment	10
Recruitment visit	100
Teleconsultation with a cardiologist	50
Remuneration of a primary care physician, for conducting follow-up and final visits	200
Televisits with a primary care physician	25
Visits with a cardiologist	250
Final teleconsultation with a cardiologist	50
Primary care nurse	150
Operation of the information and registration desk at each primary healthcare facility during the medical event—recruitment stage	59.04
Telemonitoring	60.61
Helpline	11.66
Distribution	6.14
License for telemedicine platform	0.81
Scales	41.82
Blood pressure monitors	81
Programming of scales and blood pressure monitors and configuration for the telemedicine platform	14.76
Preparation of starter kits for patients ready to be dispensed by the medical personnel	49.20
Leaflets/Posters	0.37
Social media advertising and information	0.65
Maintaining an information profile on social media	1.08
**Total**	**1162.12**

**Table 5:** Total study pool cost comparison between BSC and BSC + T (PLN).

	3-Month BSC Costs	3-Month BSC + T Costs	Difference (BSC + T − BSC)
Hospitalization	250,283.1	29,010.8	−221,272.3
Medicines	131,324.8	153,556.1	22,231.3
Outpatient specialist care and primary care	10,427.2	0	−10,427.2
Telemedical intervention		538,841.6	538,841.6
Total	392,035.1	721,408.5	329,373.4

## Data Availability

The data are contained within the article.
